# Structural insights and *in vitro* reconstitution of membrane targeting and activation of human PI4KB by the ACBD3 protein

**DOI:** 10.1038/srep23641

**Published:** 2016-03-24

**Authors:** Martin Klima, Dániel J. Tóth, Rozalie Hexnerova, Adriana Baumlova, Dominika Chalupska, Jan Tykvart, Lenka Rezabkova, Nivedita Sengupta, Petr Man, Anna Dubankova, Jana Humpolickova, Radim Nencka, Vaclav Veverka, Tamas Balla, Evzen Boura

**Affiliations:** 1 Institute of Organic Chemistry and Biochemistry AS CR, v.v.i., Flemingovo nam. 2., 16610 Prague 6, Czech Republic; 2 Section on Molecular Signal Transduction, Program for Developmental Neuroscience, NICHD, NIH, Bethesda, MD 20892, USA; 3 Laboratory of Biomolecular Research, Department of Biology and Chemistry, Paul Scherrer Institute, 5232, Villigen PSI, Switzerland; 4 Institute of Microbiology AS CR, v.v.i., Videnska 1083, Prague, Czech Republic; 5 Department of Biochemistry, Faculty of Science, Charles University in Prague, Hlavova 8, Prague, Czech Republic

## Abstract

Phosphatidylinositol 4-kinase beta (PI4KB) is one of four human PI4K enzymes that generate phosphatidylinositol 4-phosphate (PI4P), a minor but essential regulatory lipid found in all eukaryotic cells. To convert their lipid substrates, PI4Ks must be recruited to the correct membrane compartment. PI4KB is critical for the maintenance of the Golgi and trans Golgi network (TGN) PI4P pools, however, the actual targeting mechanism of PI4KB to the Golgi and TGN membranes is unknown. Here, we present an NMR structure of the complex of PI4KB and its interacting partner, Golgi adaptor protein acyl-coenzyme A binding domain containing protein 3 (ACBD3). We show that ACBD3 is capable of recruiting PI4KB to membranes both *in vitro* and *in vivo*, and that membrane recruitment of PI4KB by ACBD3 increases its enzymatic activity and that the ACBD3:PI4KB complex formation is essential for proper function of the Golgi.

Phosphatidylinositol 4-kinase beta (PI4KB, also known as PI4K IIIβ) is a soluble cytosolic protein yet its function is to phosphorylate membrane lipids. It is one of four human PI4K enzymes that phosphorylate phosphatidylinositol (PI) to generate phosphatidylinositol 4-phosphate (PI4P)^1^,^2^. PI4P is an essential lipid found in various membrane compartments including the Golgi and trans-Golgi network (TGN), the plasma membrane and the endocytic compartments. In these locations, PI4P plays an important role in cell signaling and lipid transport, and serves as a precursor for higher phosphoinositides or as a docking site for clathrin adaptor or lipid transfer proteins^3^. A wide range of positive-sense single-stranded RNA viruses (+RNA viruses), including many that are important human pathogens, hijack human PI4KA or PI4KB enzymes to generate specific PI4P-enriched organelles called membranous webs or replication factories. These structures are essential for effective viral replication^4^. Recently, highly specific PI4KB inhibitors were developed as potential antivirals^5^,^6^.

PI4K kinases must be recruited to the correct membrane type to fulfill their enzymatic functions. Type II PI4Ks (PI4K2A and PI4K2B) are heavily palmitoylated and thus behave as membrane proteins^7^. In contrast, type III PI4Ks (PI4KA and PI4KB) are soluble cytosolic proteins that are recruited to appropriate membranes indirectly via protein-protein interactions. The recruitment of PI4KA to the plasma membrane by EFR3 and TTC7 is relatively well understood even at the structural level^8^,^9^, but, the actual molecular mechanism of PI4KB recruitment to the Golgi is still poorly understood.

Acyl-coenzyme A binding domain containing protein 3 (ACBD3, also known as GCP60 and PAP7) is a Golgi resident protein^10^. Its membrane localization is mediated by the interaction with the Golgi integral protein golgin B1/giantin^11^. ACBD3 functions as an adaptor protein and signaling hub across cellular signaling pathways. ACBD3 can interact with a number of proteins including golgin A3/golgin-160 to regulate apoptosis^12^, Numb proteins to control asymmetric cell division and neuronal differentiation^13^, metal transporter DMT1 and monomeric G protein Dexras1 to maintain iron homeostasis^14^, and the lipid kinase PI4KB to regulate lipid homeostasis^15^,^16^. ACBD3 has been also implicated in the pathology of neurodegenerative diseases such as Huntington’s disease due to its interactions with a polyglutamine repeat-containing mutant huntingtin and the striatal-selective monomeric G protein Rhes/Dexras2^17^. ACBD3 is a binding partner of viral non-structural 3A proteins and a host factor of several picornaviruses including poliovirus, coxsackievirus B3, and Aichi virus^15^,^16^.

We present a biochemical and structural characterization of the molecular complex composed of the ACBD3 protein and the PI4KB enzyme. We show that ACBD3 can recruit PI4KB to model membranes as well as redirect PI4KB to cellular membranes where it is not naturally found. Our data also show that ACBD3 regulates the enzymatic activity of PI4KB kinase through membrane recruitment rather than allostery.

## Results

### ACBD3 and PI4KB interact with 1:1 stoichiometry with submicromolar affinity

**Figure 1:**
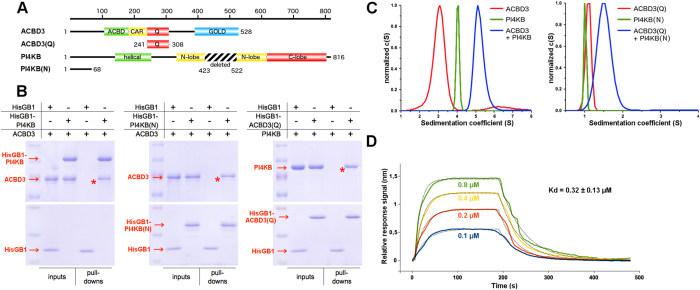
Biochemical characterization of the ACBD3:PI4KB complex. (**A**) Schematic representation of the ACBD3 and PI4KB constructs used for the experiments. ACBD3 contains the acyl-CoA binding domain (ACBD), charged amino acids region (CAR), glutamine rich region (Q), and Golgi dynamics domain (GOLD)^16^. PI4KB is composed of the N-terminal region, helical domain, and kinase domain which can be divided into N- and C-terminal lobes^1^. (**B**) *In vitro* pull-down assay. Pull-down assays were performed using NiNTA-immobilized N-terminal His_6_GB1-tagged proteins as indicated and untagged full-length PI4KB or ACBD3. The inputs and bound proteins were analyzed on SDS gels stained with Coomassie Blue. The asterisks mark the bands corresponding to specific interactions. Cropped gels ran the same experimental conditions are shown. Please, see SI Fig. 9 for original full-length gels. (**C**) Analytical Ultracentrifugation. AUC analysis of the ACBD3:PI4KB full-length complex at the concentration of 5 μM (both proteins, left panel) and ACBD3 Q domain: PI4KB N terminal region complex at the concentration of 35 μM (both proteins, right panel). (**D**) Surface plasmon resonance. SPR analysis of the PI4KB binding to immobilized ACBD3. Sensorgrams for four concentrations of PI4KB are shown.

In order to verify the interactions between ACBD3 and PI4KB we expressed and purified both proteins. To increase yields of bacterial expression the intrinsically disordered region of PI4KB (residues 423–522)^18^ was removed (Fig. 1A). This internal deletion does not significantly affect the kinase activity^19^(SI Fig. 1A) or interaction with ACBD3 (SI Fig. 1B,C). In an *in vitro* binding assay, ACBD3 co-purified with the NiNTA-immobilized N-terminal His_6_GB1-tagged PI4KB (Fig. 1B, left panel), suggesting a direct interaction. Using a mammalian two-hybrid assay Greninger and colleagues^20^ localized this interaction to the Q domain of ACBD3 (named according to its high content of glutamine residues) and the N-terminal region of PI4KB preceding its helical domain. We expressed the Q domain of ACBD3 (residues 241–308) and the N-terminal region of PI4KB (residues 1–68) in *E. coli* and using purified recombinant proteins, we confirmed that these two domains are sufficient to maintain the interaction (Fig. 1B, middle and right panel).

Because it has been reported that ACBD3 can dimerize in a mammalian two-hybrid assay, we were interested in determining the stoichiometry of the ACBD3:PI4KB protein complex. The sedimentation coefficients of ACBD3 and PI4KB alone, or ACBD3:PI4KB complex were determined by analytical ultracentrifugation and found to be 3.1 S, 4.1 S, and 5.1 S. These values correspond to molecular weights of approximately 55 kDa, 80 kDa, and 130 kDa, respectively. This result suggests that both proteins are monomeric and the stoichiometry of the ACBD3: PI4KB protein complex is 1:1 (Fig. 1C, left panel). Similar results were obtained for the complex of the Q domain of ACBD3 and the N-terminal region of PI4KB (Fig. 1C, right panel). We also determined the strength of the interaction between recombinant full length ACBD3 and PI4KB using surface plasmon resonance (SPR). SPR measurements revealed a strong interaction with a Kd value of 320 +/−130 nM (Fig. 1D, SI Fig. 1D). We concluded that ACBD3 and PI4KB interact directly through the Q domain of ACBD3 and the N-terminal region of PI4KB forming a 1:1 complex with a dissociation constant in the submicromolar range.

### Structural analysis of the ACBD3:PI4KB complex

Full length ACBD3 and PI4KB both contain large intrinsically disordered regions that impede crystallization^21^. We used hydrogen-deuterium exchange mass spectrometry (HDX-MS) analysis of the complex to determine which parts of the complex are well folded (SI Fig. 2). However, we were unable to obtain crystals even when using significantly truncated constructs that included only the ACBD3 Q domain and the N-terminal region of PI4KB.

For this reason, we produced an isotopically labeled ACBD3 Q domain and isotopically labeled ACBD3 Q domain:PI4KB N-terminal region protein complex and used NMR spectroscopy for structural characterization. As the N-terminal region protein complex was prepared by co-expression of both proteins, the samples consisted of an equimolar mixture of two uniformly ^15^N/^13^C labelled molecules. Comprehensive backbone and side-chain resonance assignments for the free ACBD3 Q domain and the complex, as illustrated by the 2D ^15^N/^1^H HSQC spectra (SI Figs 3 and 4), were obtained using a standard combination of triple-resonance experiments, as described previously. Backbone amide signals (^15^N and ^1^H) for the free ACBD3 Q domain were nearly completely assigned apart from the first four N-terminal residues (Met^1^-Lys^4^) and Gln^44^. Over 93% of non-exchangeable side-chain signals were assigned for the free ACBD3 Q domain. Apart from the four N-terminal residues, the side-chain assignments were missing for Gln^25^ (H^g3^), Gln^40^ (H^a^/H^b^/H^g^), Gln44 (H^a^/H^b^/H^g^) and Gln^48^ (H^g^) mainly due to extensive overlaps within the spectral regions populated by highly abundant glutamine side-chain resonances. The protein complex yielded relatively well resolved spectra (SI Fig. 4) that resulted in assignment of backbone amide signals for all residues apart from Gln^2^ (ACBD3) and Ala^2^ (PI4KB). Assignments obtained for non-exchangeable side-chain signals were over 99% complete. The essentially complete ^15^N, ^13^C and ^1^H resonance assignments allowed automated assignment of the NOEs identified in the 3D ^15^N/^1^H NOESY-HSQC and ^13^C/^1^H HMQC-NOESY spectra that were subsequently used in structural calculation. Structural statistics for the final water-refined sets of structures are shown in SI Table 1.

**Figure 2:**
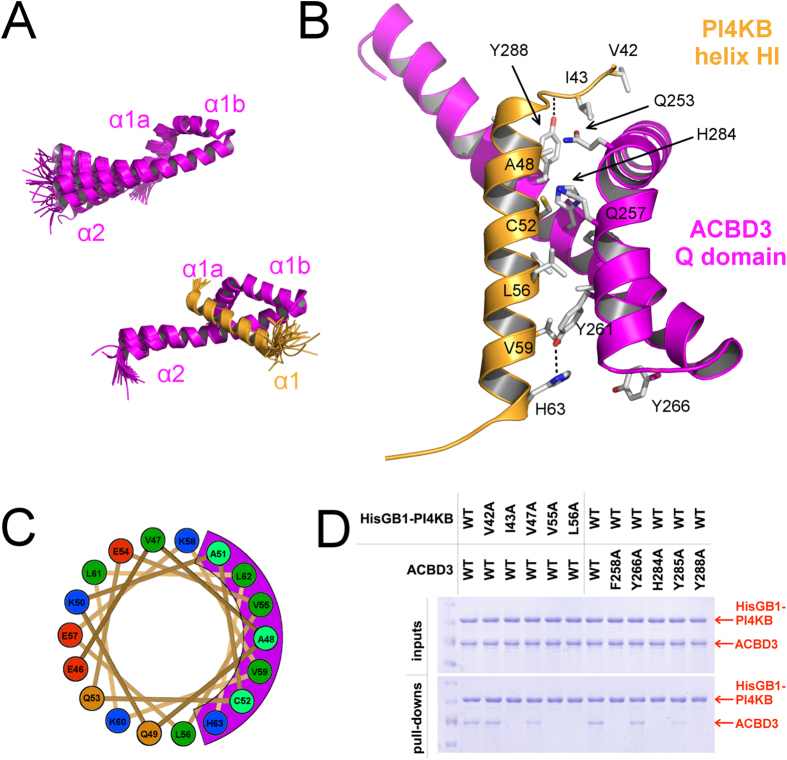
Structural analysis of the ACBD3:PI4KB complex. (**A**) Overall structure of the ACBD3 Q domain by itself and in complex with the PI4KB N-terminal region. Superposition of the 30 converged structures obtained for the Q domain (top) and the 45 converged structures obtained for the complex (bottom), with only the folded part of PI4KB shown (see SI Fig. 2 for the complete view). (**B**) Detailed view of the complex. The interaction is facilitated by only two hydrogen bonds (ACBD3 Tyr^261^: PI4KB His^63^ and ACBD3 Tyr^288^: PI4KB Asp^44^), while the hydrophobic surface of the kinase helix nests in the ACBD3 Q domain. ACBD3 is shown in magenta and PI4KB in orange. (**C**) Top view of the kinase helix. The kinase helix is amphipathic and its hydrophobic surface overlaps with the ACBD3 binding surface (shown in magenta). Strong and weak hydrophobes are in green and cyan respectively, basic residues in blue, acidic residues in red and nonpolar hydrophilic residues in orange. (**D**) Pull-down assay with a NiNTA-immobilized N-terminally His6GB1-tagged PI4KB kinase and untagged ACBD3 protein. Wild type proteins and selected point mutants of both PI4KB and ACBD3 were used. Inputs and bound proteins were analyzed on SDS gels and stained with Coomassie Blue. Cropped gels ran the same experimental conditions are shown. Please, see SI Fig. 9 for original full-length gels.

This structure revealed that the Q domain forms a two helix hairpin. The first helix bends sharply over the second helix and creates a fold resembling a three helix bundle that serves as a nest for one helix of the PI4KB N-terminus (residues 44–64, from this point on referred to as the kinase helix) (Fig. 2A). Preceding the kinase helix are three ordered residues (Val^42^, Ile^43^, and Asp^44^) that also contribute to the interaction (Fig. 2B). The remaining part of the PI4KB N-termini, however, is disordered (SI Fig. 5). Almost all of the PI4KB:ACBD3 interactions are hydrophobic with the exception of hydrogen bonds between the side chains of ACBD3 Tyr^261^ and PI4KB His^63^, and between the sidechain of ACBD3 Tyr^288^ and the PI4KB backbone (Asp^44^) (Fig. 2B). Interestingly, we noted that the PI4KB helix is amphipathic and its hydrophobic surface leans on the Q domain (Fig. 2C).

To corroborate the structural data, we introduced a number of point mutations and validated their effect on complex formation using an *in vitro* pull-down assay (Fig. 2D). Wild type ACBD3 protein co-purified together with the NiNTA-immobilized His_6_-tagged wild type PI4KB as well as with the PI4KB V^42^A and V^47^A mutants, but not with mutants within the imminent binding interface (I^43^A, V^55^A, L^56^A). As predicted, wild type PI4KB interacted with the ACBD3 Y^266^A mutant and slightly with the Y^285^A mutant, but not with the F^258^A, H^284^A, and Y^288^A mutants (Fig. 2D).

### ACBD3 efficiently recruits the PI4KB enzyme to membranes

**Figure 3:**
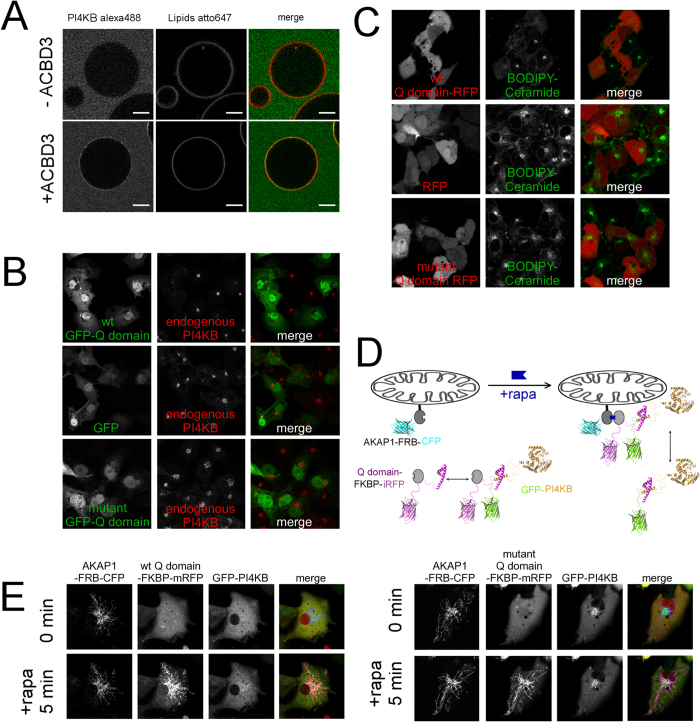
ACBD3 is sufficient to recruit the PI4KB kinase to membranes. (**A**) GUVs recruitment assay. Top – Virtually no membrane bound kinase was observed when 600 nM PI4KB was added to the GUVs. Bottom – in the presence of 600 nM GUV tethered ACBD3 a significant signal of the kinase is detected on the surface of GUVs. (**B**) Golgi displacement experiment. Upper panel: ACBD3 Q domain fused to GFP was overexpressed and the endogenous PI4KB was immunostained. Middle panel: The same experiment performed with GFP alone. Lower panel: The same experiment performed with mutant Q domain (F^258^A, H^284^A, Y^288^A) that does not bind the PI4KB. (**C**) ACBD3 Q domain overexpression inhibits ceramide transport to Golgi – COS-7 cells transfected with wild-type ACBD3 Q domain-FKBP-mRFP were loaded with 0.05 μM Bodipy FL-Ceramide for 20 min, then washed and depicted after 20 min. Middle panel – The same experiment performed with mRFP-FKBP alone. Lower panel – The same experiment performed with mutant Q domain (F^258^A, H^284^A, Y^288^A) that does not bind the PI4KB. (**D**) Scheme of the mitochondria recruitment experiment. – The AKAP1-FRB-CFP construct is localized at the outer mitochondrial membrane, while the GFP-PI4KB and Q domain-FKBP-mRFP constructs are localized in the cytoplasm where they can form a complex. Upon addition of rapamycin the Q domain-FKBP-mRFP construct translocates to the mitochondria and takes GFP-PI4KB with it. (**E**) Mitochondria recruitment experiment. Left – cells transfected with AKAP1-FRB-CFP, GFP-PI4KB and wild-type Q domain-FKBP-mRFP constructs before and five minutes after addition of rapamycin. Right – The same experiment performed using the H^264^A Q domain mutant.

We next sought to determine if the ACBD3:PI4KB interaction drives membrane localization of the PI4KB enzyme. To do this, we first established an *in vitro* membrane recruitment system using Giant Unilamellar Vesicles (GUVs) containing the PI4KB substrate – the PI lipid. We observed that PI4KB kinase was not membrane localized when added to the GUVs at 600 nM concentration, whereas non-covalent tethering of ACBD3 to the surface of the GUVs, using the His_6_ tag on ACBD3 and the DGS-NTA (Ni) lipid, led to efficient PI4KB membrane localization (Fig. 3A).

We hypothesized that if ACBD3 is one of the main Golgi localization signals for PI4KB, overexpression of the Q domain should decrease the amount of the endogenous kinase on the Golgi. Indeed, we observed loss for endogenous PI4KB signal on the Golgi in cells overexpressing the GFP – Q domain construct (Fig. 3B upper panel). We attribute the loss of signal to the immunostaining protocol-the kinase that is not bound to Golgi is lost during the permeabilization step and hence the “disappearance” of the signal because overexpression of GFP alone or a non-binding Q domain mutant has no effect on the localization of the endogenous PI4KB (Fig. 3B). Given this result, overexpression of the Q domain should also interfere with the PI4KB dependent Golgi functions. Ceramide transport and accumulation in Golgi is a well-known PI4KB dependent process^22^. We have used fluorescently labeled ceramide and analyzed its trafficking in non-transfected cells and cell overexpressing the Q domain. As expected, the Golgi accumulation of ceramide was not observed in cells expressing the wt Q domain while cells expressing RFP or the mutant Q domain accumulated ceramide normally (Fig. 3C) suggesting that ACBD3:PI4KB complex formation is crucial for the normal function of Golgi.

We further analyzed the function of ACBD3:PI4KB interaction in membrane recruitment of PI4KB in living cells using fluorescently tagged proteins. We used the rapamycin-inducible heteromerization of FKBP12 (FK506 binding protein 12) and FRB (fragment of mTOR that binds rapamycin) system^23^,^24^. We fused the FRB to residues 34–63 of the mitochondrial localization signal from mitochondrial A-kinase anchor protein 1 (AKAP1) and CFP. The ACBD3 Q domain was then fused to FKBP12 and mRFP (Fig. 3D). We analyzed localization of the ACBD3 Q domain and GFP – PI4KB before and after the addition of rapamycin. As a control we used H^284^A mutant of the ACBD3 Q domain that does not significantly bind PI4KB kinase. In every case the ACDB3 Q domain was rapidly (within 5 minutes) recruited to the mitochondrial membrane upon addition of rapamycin, but only the wild-type protein effectively directed the kinase to the mitochondria (Fig. 3E, Movie 1 and 2). Notably, we observed that when the GFP-PI4KB kinase is co-expressed with the wild-type ACDB3 Q domain it loses its typical Golgi localization (Fig. 3E upper panel). However, PI4KB retains it Golgi localization when co-expressed with the non-interacting Q domain mutant (Fig. 3E lower panel).

### ACBD3 increases PI4KB enzymatic activity by recruiting PI4KB to close vicinity of its substrate

**Figure 4:**
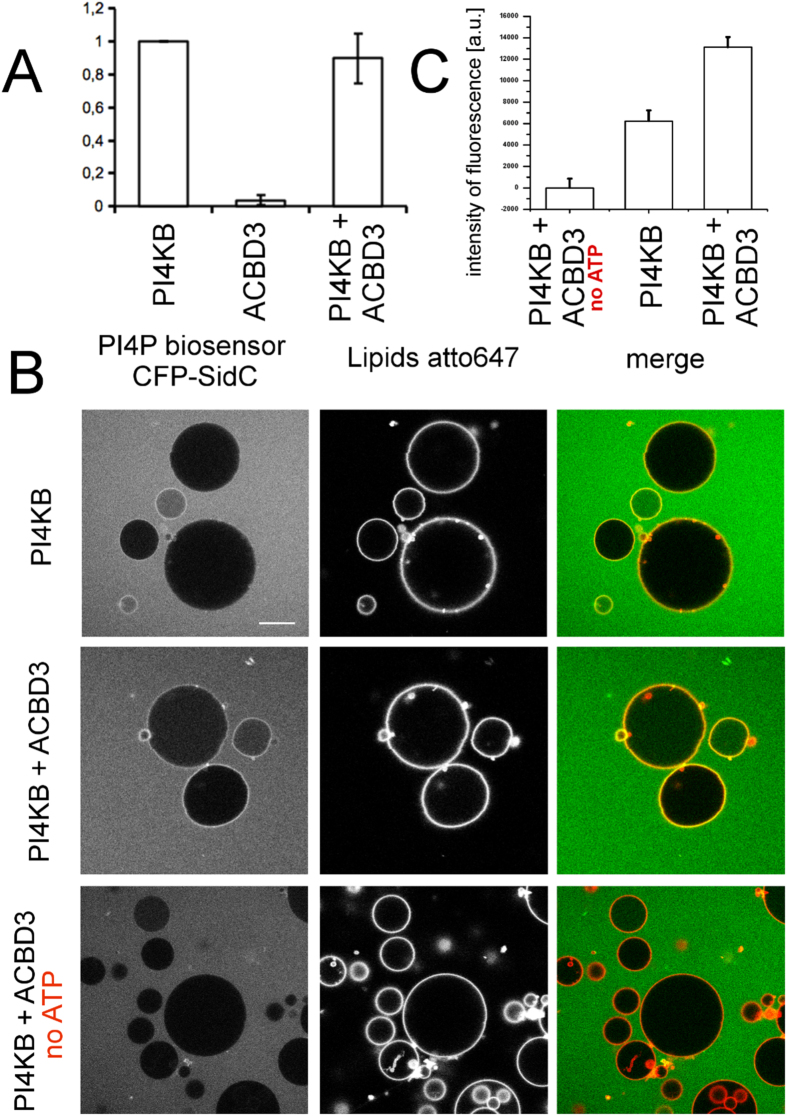
ACBD3 indirectly increases the activity of PI4KB. (**A**) Micelles-based kinase assay – PI in TX100 micelles was used in a luminescent kinase assay and the production of PI4P was measured. Bar graph presents the mean values of PI4P generated in the presence of the proteins as indicated, normalized to the amount of PI4P generated by PI4KB alone. Error bars are standard errors of the mean (SEM) based on three independent experiments. (**B**) GUV-based phosphorylation assay – GUVs containing 10% PI were used as a substrate and the production of PI4P was measured using the CFP-SidC biosensor. (**C**)–Quantification of the GUV phosphorylation assay – Mean membrane fluorescence intensity of the PI4P reporter (SidC-label) under different protein/ATP conditions. The mean membrane intensity value is relative to the background signal and the difference between the membrane and background signal in the reference system lacking ATP. The error bars stand for SEM based on three independent experiments (also SI Fig. 6).

To test whether ACBD3 can stimulate PI4KB kinase enzymatic activity we performed a standard luminescent kinase assay using PI-containing micelles as the substrate^25^. We observed no effect on the kinase activity of PI4KB (Fig. 4A) suggesting that ACBD3 does not directly affect the enzyme (e.g. induction of a conformation change). However, *in vivo* ACBD3 is located at the Golgi membranes, whereas in this experiment, ACBD3 was located in the solution and PI is provided as micelles. We therefore designed a more physiologically relevant experiment. For this, we again turned to the GUV system with ACBD3 localized to the GUV membrane. The GUVs contained 10% PI to serve as a substrate for PI4KB kinase. The buffer also contained CFP-SidC, which binds to PI4P with nanomolar affinity^26^. This enabled visualization of the kinase reaction using a confocal microscope. We compared the efficiency of the phosphorylation reaction of the kinase alone with that of kinase recruited to the surface of the GUVs by ACBD3. Reaction was also performed in the absence of ATP as a negative control (Fig. 4B). These experiments showed that PI4KB enzymatic activity increases when ACBD3 is membrane localized (Fig. 4C, SI Fig. 6). We conclude that enzyme activation proceeds through a membrane recruitment mechanism.

## Discussion

Membrane recruitment of PI4KB enzyme is crucial to ensure its proper function at the Golgi and TGN. However, the molecular mechanism and structural basis for PI4KB interaction with the membrane is poorly understood. In principle, any of the binding partners of PI4KB could play a role in membrane recruitment. To date, several PI4KB interacting proteins have been reported, including the small GTPases Rab11 and Arf1, the Golgi resident acyl-CoA binding domain containing 3 (ACBD3) protein, neuronal calcium sensor-1 (NCS-1 also known as frequenin in yeast) and the 14-3-3 proteins.

The monomeric G protein Rab11 binds mammalian PI4KB through the helical domain of the kinase. Although Rab11 does not appear to be required for recruitment of PI4KB to the Golgi, PI4KB is required for Golgi recruitment of Rab11^27^. Arf1, the other small GTP binding protein, is known to influence the activity and localization of PI4KB, but it does not appear to interact directly with PI4KB (our unpublished data). The yeast homologue of NCS1 called frequenin has been shown to interact with Pik1p, the yeast orthologue of PI4KB and regulate its activity and perhaps its membrane association^28^, but the role of NCS-1 in PI4KB recruitment in mammalian cells is unclear. NCS-1 is an N-terminally myristoylated protein that participates in exocytosis. It is expressed only in certain cell types, suggesting that if it contributes to PI4KB membrane recruitment, it does so in a tissues specific manner^29^. The interaction of PI4KB with 14-3-3 proteins, promoted by phosphorylation of PI4KB by protein kinase D, influences the activity of PI4KB by stabilizing its active conformation. However, 14-3-3 proteins do not appear to interfere with membrane recruitment of this kinase^30^. ACBD3 is a Golgi resident protein, conserved among vertebrates (SI Fig. 7), that interacts directly with PI4KB (see also SI Fig. 8 and SI Discussion), and whose genetic inactivation interferes with the Golgi localization of the kinase^31^. For these reasons we focused on the interaction of the PI4KB enzyme with the Golgi resident ACBD3 protein in this study.

**Figure 5:**
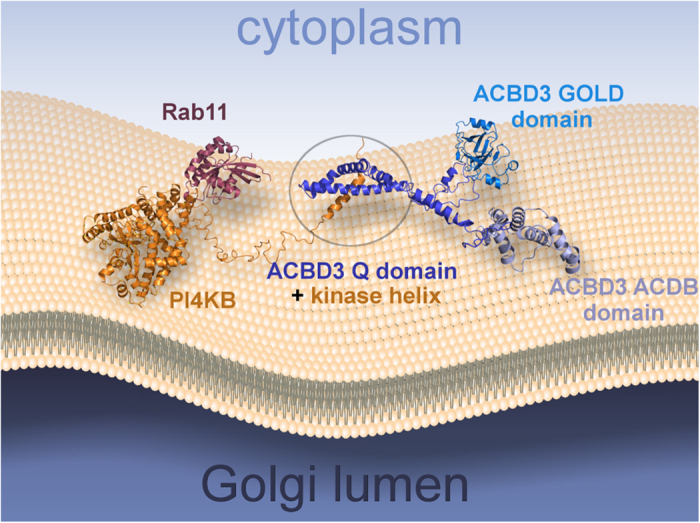
Pseudoatomic model of the PI4KB multiprotein complex assembly. PI4KB in orange, Rab11 in purple, ACBD3 in blue. The model is based on our NMR structure and a previously published crystal structure of PI4KB:Rab11 complex (PDB code 4D0L), ACBD and GOLD domain were homology modeled based on high sequence identity structures produced by the Phyre2 web server^41^. The GOLD domain is tethered to the membrane by GolginB1 (also known as Giantin)^11^ which is not shown for clarity. Intrinsically disordered linkers are modeled in an arbitrary but physically plausible conformation.

Here we present the mechanism for membrane recruitment of PI4KB by the Golgi resident ACBD3 protein. We show that these proteins interact directly with a Kd value in the submicromolar range. The interaction is sufficient to recruit PI4KB to model membranes *in vitro* as well as to the mitochondria where PI4KB is never naturally found. To understand this process at the atomic level we solved the solution structure of ACBD3:PI4KB sub complex (Fig. 1A) and found that the PI4KB N-terminal region contains a short amphipatic helix (residues 44–64) that binds the ACBD3 Q domain. The Q domain adopts a helical hairpin fold that is further stabilized upon binding the kinase helix (Fig. 2A). Our data strongly suggest that formation of the complex does not directly influence the catalytic abilities of the kinase but experiments with model membranes revealed that ACBD3 enhances catalytic activity of the kinase by a recruitment based mechanism; it recruits the kinase to the membrane and thus increases the local concentration of the substrate in the vicinity of the kinase. Based on our and previously published structures we built a pseudoatomic model of PI4KB multi-protein assembly on the membrane (Fig. 5) that illustrates how the enzyme is recruited and positioned towards its lipidic substrate and how it in turn recruits Rab11.

+RNA viruses replicate at specific PI4P-enriched membranous compartments. These are called replication factories (because they enhance viral replication) or membranous webs (because of their appearance under the electron microscope)^4^. To generate replication factories, viruses hijack several host factors including the PI4K kinases to secure high content of the PI4P lipid. Non-structural 3A proteins from many picornaviruses from the Enterovirus (e.g. poliovirus, coxsackievirus-B3, rhinovirus-14) and Kobuvirus (e.g. Aichi virus-1) genera directly interact with ACBD3^15^,^16^. Our data suggest that they could do this via 3A:ACBD3:PI4KB complex formation. The structure of the ACBD3 Q domain and the kinase helix described here provides a novel opportunity for further research on the role of ACBD3, PI4KB, and the ACBD3:PI4KB interaction in picornaviral replication. This could eventually have implications for therapeutic intervention to combat picornaviruses-mediated diseases ranging from polio to the common cold.

## Materials and Methods

### Plasmid construction, protein expression, and purification

All proteins used in this study were recombinant and were expressed in *E. coli* using previously developed protocols^32^,^33^. Briefly, full-length human ACBD3 (UniProtKB entry Q9H3P7) and PI4KB (UniProtKB entry Q9UBF8, isoform 1) lipid kinase and their deletion mutants were cloned into a previously modified pRSFD vector (Novagen) that already contained an N-terminal 6xHis tag followed by a GB1 solubility tag and TEV protease cleavage site. Mutations were generated using the Phusion Site-Directed Mutagenesis Kit (Thermo Scientific). The plasmids used are listed in the SI (SI Table 2). The proteins were expressed in *E. coli* BL21 Star cells as previously described^5^,^34^. Upon overnight expression in autoinduction media bacterial cells were harvested and lysed in lysis buffer (50 mM Tris pH 8, 300 mM NaCl, 3 mM β-mercaptoethanol, 20 mM imidazole, 10% glycerol). The lysate was incubated with the Ni-NTA resin (Macherey-Nagel) and then extensively washed with the lysis buffer. The protein was eluted with the lysis buffer supplemented with 300 mM imidazole. When appropriate, tags were removed with TEV protease, and the protein was further purified using the size exclusion chromatography on Superdex 75 or Superdex 200 columns (GE Healthcare) in SEC buffer (10 mM Tris pH 8, 200 mM NaCl, 3 mM β-ME). Proteins were concentrated to 1–5 mg/ml (measured spectroscopically) and stored at −80 °C until needed.

### *In vitro* pull-downs

Ni-NTA sepharose beads (Macherey-Nagel) were mixed with both binding partners (one of which was tagged with an N-terminal 6xHis tag) at a final concentration of 1 μM in a final volume of 200 μL binding buffer (30 mM Tris pH 8, 200 mM NaCl, 10 mM imidazole, and 1 mM TCEP). After 30 min incubation at 4 °C the beads were washed twice with 200 μL of the binding buffer, and total protein was directly eluted with the Laemmli sample buffer and analyzed by SDS-PAGE.

### SPR (Surface plasmon resonance) and AUC (Analytical ultracentrifugation)

PI4KB was chip-immobilized as detailed in the SI. Afterwards, the ACBD3 protein was injected in a series of concentrations for 3 min and then dissociation was monitored for another 5 min. The data were fit to a single-exponential model. Rate constants of association and dissociation were obtained by fitting the observed change in resonance signal using the following equations:


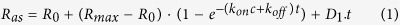



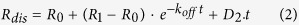


where c is the protein concentration, t is time, k_on_ is the association rate constant, k_off_ is the dissociation rate constant, D_1_ and D_2_ are the linear drift terms, and R_as_, R_dis_, R_0_, R_1_, and R_max_ are corresponding changes in the relative response signal.

AUC was used to perform sedimentation velocity experiments using a ProteomeLab XL-I Beckman Coulter analytical ultracentrifuge equipped with an AN50Ti rotor. All measurements were performed in 10 mM Tris pH 8, 200 mM NaCl, and 3 mM β-mercaptoethanol at 20 °C and 48000 rpm. All data were collected using an absorbance (230 nm and 280 nm) optical system. Data analysis was performed with the SEDFIT package^35^ and data were analyzed using a sedimentation coefficient distribution model c(s).

### *In vitro* kinase assay

*In vitro* kinase activity was measured using a bioluminescent ADP-Glo assay (Promega) as described previously^36^. Briefly, reactions were carried out in a total volume of 5 μL in 384-well plates by diluting the indicated amounts of the PI4KB enzyme and/or ACBD3 protein into the kinase buffer (20 mM Tris pH 7.5, 5 mM MgCl_2_, 0.2% Triton-X100, 0.1 mg/mL BSA, 2 mM DTT, 50 μM phosphatidylinositol). Reaction was initiated by adding ATP to a final concentration of 100 μM. Samples were incubated for 60 min at 25 °C and the amount of hydrolyzed ATP was measured according to the manufacturer’s protocol using a TECAN infinite M 1000 plate reader.

### NMR spectroscopy

NMR spectra were acquired at 25 °C on a 600 MHz and 850 MHz Bruker Avance spectrometers, both of which were equipped with a triple-resonance (^15^N/^13^C/^1^H) cryoprobe. The sample volume was 0.35 mL, with a 280 μM concentration for the free Q domain and a 470 μM concentration for the ACBD3:PI4KB complex in the NMR buffer (25 mM sodium phosphate pH 6.5, 100 mM NaCl, 1 mM TCEP, 0.01% NaN_3_), 5% D_2_O/95% H_2_O. A series of double- and triple-resonance spectra^37^,^38^ were recorded to determine essentially complete sequence-specific resonance backbone and side-chain assignments. Constraints for ^1^H-^1^H distance required to calculate the structure of free Q domain and ACBD3:PI4KB complex were derived from 3D ^15^N/^1^H NOESY-HSQC and ^13^C/^1^H NOESY-HMQC, which were acquired using a NOE mixing time of 100 ms.

The families of converged structures for the ACBD3:PI4KB complex and free Q domain were calculated using standard software as detailed in the SI. The structures with the lowest total energy were selected and validated. The statistics for the resulting structures are summarized in SI Table 1.

### Protein labeling with fluorescent dyes

PI4KB was labeled on native cysteine residues. Briefly, pure recombinant protein was incubated overnight at 4 °C with a 3x molar excess of Alexa 488 C5 maleimide (Life Technologies). The reaction was quenched by adding 10 mM β-mercaptoethanol (βME) and the protein was repurified by size exclusion chromatography.

### Giant Unilamellar Vesicle Preparation and Imaging

Giant Unilamellar Vesicles (GUVs) composed of POPC (54.9 mol %), POPS (10 mol %), cholesterol (20 mol %), PI (10 mol %), DGS-NTA(Ni) [1,2-dioleoyl-sn-glycero-3-[(N-(5-amino-1-carboxypentyl)iminodiacetic acid)succinyl] (nickel salt) ] (5 mol %) (Avanti Polar lipids), and ATTO647N-DOPE (0.1 mol %) (ATTO-TEC GmbH) were prepared by electroformation as described previously^39^, please see SI.

### Live Cell Imaging

COS-7 cells were plated onto 29-mm-diameter poly-L-Lysine coated glass bottom dishes (*In Vitro* Scientific) at 100,000 cells/well density and transfected using the Lipofectamine2000 reagent (Invitrogen) with plasmid DNAs (0.5–1 mg/well) according to manufacturer’s instructions. The plasmids are described in SI Table 2. 24 hr post transfection, COS-7 cells were washed with a modified Krebs-Ringer solution (10 mM Na-HEPES pH 7.4, 120 mM NaCl, 4.7 mM KCl, 2 mM CaCl_2_, 0.7 mM MgSO_4_, 10 mM glucose) in the same dish and were imaged using an LSM 710 confocal microscope (Carl Zeiss MicroImaging) with a 63 × 1.4-numerical-aperture planapochromatic objective. For ceramide uptake experiments, COS-7 cells were loaded with 0.05 μM BODIPY^®^ FL C5-Ceramide (Molecular Probes, ThermoFisher Scientific) complexed with BSA in modified Krebs-Ringer solution at room temperature for 20 min. Cells were then washed three times and imaged using the above mentioned settings.

### Immunofluorescent imaging

COS-7 cells were plated onto 25-mm-diameter poly-L-Lysine coated circular glass coverslips in six-well plates (100,000 cells/well), and transfected using the Lipofectamine2000 reagent (Invitrogen) with plasmid DNAs (0.5–1 mg/well) according to manufacturer’s instructions. Twenty four hours post transfection, cells were washed with PBS, fixed with 4% paraformaldehyde, stained with mouse anti-PI4KB primary antibody (BD Transduction Laboratories, 1:500 dilution) and then after washing with PBS stained with Alexa Fluor 647 conjugated donkey anti-mouse secondary antibody (Molecular Probes, ThermoFisher Scientific, 1:500 dilution). Cover slips were mounted and observed with the above mentioned microscopy settings.

### HD exchange

Hydrogen/deuterium exchange was performed as previously described^40^ with the following modifications. The exchange was done in 10 mM Tris-HCl pD 8.0, 200 mM NaCl at 20 °C. Protein concentration during the exchange was 1 μM. Aliquots (50 μL) were removed after 10, 20, 60, 120, 600, 1800, and 3600 s and the exchange was quenched by the addition of 50 μL of 0.25 M glycine-HCl pH 2.3 and rapid freezing in liquid nitrogen.

Prior to the analysis each sample was quickly thawed and injected onto an immobilized rhizopuspepsin column (bed volume 66 μL). Digestion was driven by a flow of 0.4% formic acid in water at a flow rate of 100 μL/min (LC-20AD pump, Shimadzu). The resulting peptides were trapped and desalted online on a peptide microtrap (Optimize Technologies). After a desalting step (3 min), peptides were separated using a linear gradient of 10–25% buffer B for 2 min, followed by a quick jump to 99% buffer B (buffer A = 0.4% formic acid/2% acetonitrile in water; buffer B = 95% acetonitrile/0.4% formic acid in water). The outlet of the LC system was interfaced to an electrospray ionization source of a Fourier transform ion cyclotron resonance mass spectrometer (12 T SolariX XR, Bruker Daltonics). Exchange was followed on 32 peptides from PI4KB (N) and 26 peptides from ACBD3(Q), covering in both cases 100% of the protein sequence. Peptides were identified by LC-MS/MS and MASCOT search against a database containing the sequences of the studied proteins. Data from H/D exchange were analyzed by program DeutEx written in the laboratory (unpublished).

## Additional Information

**Accession codes:** The structures and assigned chemical shifts for the free Q domain and the ACBD3:PI4KB complex were deposited in PDB database under accession codes 2N72 and 2N73, and BMRB database under accession codes 25790 and 25791.

**How to cite this article**: Klima, M. *et al*. Structural insights and *in vitro* reconstitution of membrane targeting and activation of human PI4KB by the ACBD3 protein. *Sci. Rep.* **6**, 23641; doi: 10.1038/srep23641 (2016).

## Supplementary Material

Supplementary Information

Supplementary Video 1

Supplementary Video 2
