# Supplementary material for: Structural insights and in vitro reconstitution of membrane targeting and activation of human PI4KB by the ACBD3 protein
Source: Sci Rep. 2016 Mar 24;6:23641. doi: 10.1038/srep23641 (PMC4806292; doi:10.1038/srep23641)
Supplement: Supplementary Information [file srep23641-s1.pdf]

Structural insights and in vitro reconstitution of membrane targeting and activation of human PI4KB by the ACBD3 protein

Supplementary Information

Martin Klima[1], Daniel Toth[2], Rozalie Hexnerova[1], Adriana Baumlova[1], Dominika Chalupska[1], Jan Tykvart[1], Lenka Rezabkova[3], Nivedita Sengupta[2], Petr Man[4,5], Anna Dubankova[1], Jana Humpolickova[1], Radim Nencka[1], Vaclav Veverka[# 1], Tamas Balla[# 2], Evzen Boura[1,*]

[1]Institute of Organic Chemistry and Biochemistry AS CR, v.v.i., Flemingovo nam. 2., 166 10 Prague 6, Czech Republic

[2]Section on Molecular Signal Transduction, Program for Developmental Neuroscience, NICHD, NIH, Bethesda, MD 20892, USA

[3]Laboratory of Biomolecular Research, Department of Biology and Chemistry, Paul Scherrer Institute, 5232, Villigen PSI, Switzerland

[4]Institute of Microbiology AS CR, v.v.i., Videnska 1083, Prague, Czech Republic

[5]Department of Biochemistry, Faculty of Science, Charles University in Prague, Hlavova 8, Prague, Czech Republic

[#] co-senior author

*correspondence to boura@uochb.cas.cz

Supplementary Discussion

The TBC1 domain family members 22A (TBC1D22A) and 22B (TBC1D22B) were recently shown to interact with the Q domain of ACBD3 [1]. Members of this family were proposed to act as GTPase activating proteins for Rab family members which are involved in vesicle trafficking. TBC1D22A and B share a high degree of homology, are Golgi membrane localized, and act as putative RabGAPs for Rab33. Given the similarity of the primary sequences of the ACBD3 binding sites of PI4KB and TBC1D22A/B (SI Fig. 8A), we were able to generate homology models of the ACBD3-TBC1D22A and ACBD3-TBC1D22B complexes (SI Fig. 8B). These models are in a good agreement with previously published observations that the ACBD3-PI4KB and ACBD3-TBC1D22A/B interactions are mutually exclusive, suggesting a possible regulatory mechanism for Golgi recruitment of the PI4KB enzyme [1].

Supplementary Materials and Methods

SPR (Surface plasmon resonance) and AUC (Analytical ultracentrifugation) – SPR measurements were performed on a four-channel SPR sensor platform (PLASMON IV) developed at the Institute of Photonics and Electronics, AS CR, Prague. A gold chip was first functionalized with alkanethiols containing carboxylic terminal groups (Prochimia) in pure ethanol, and then mounted on the prism of an SPR sensor. All experiments were performed at 25 °C at a flow rate of 30 µL/min. Activation of carboxylic terminal groups on the sensor surface was performed in situ with a solution of N-hydroxysuccinimide and N-ethyl-N-(dimethylaminopropyl)-carbodiimide hydrochloride (Biacore). Then, a 0.02 mg/mL neutravidin solution in the SA buffer (10 mM sodium acetate pH 5) was loaded, followed by a high ionic strength solution (10 mM sodium phosphate pH 7.4, 0.5 M NaCl) to wash out non-covalently bound neutravidin, and 1 M ethanolamine to deactivate residual carboxylic groups. For immobilization a 100 nM solution of an appropriate recombinant biotinylated protein in the running buffer (10 mM Tris pH 8, 200 mM NaCl, and 1 mM TCEP) was used to achieve approximately 2 nm change in the relative response signal (the protein biotinylation was achieved by co-expression of the appropriate protein tagged with an AviTag at its N-terminus with a biotin ligase BirA in E. coli as described in Kay et al. [2]). Afterwards, untagged proteins in the indicated series of concentrations in the running buffer were injected for 3 min and then the dissociation was monitored for another 5 min. The data were fit to a single-exponential model. Rate constants of association and dissociation were obtained by fitting the observed change in resonance signal using the following equations:

(1) $$R_{as} = R_0 + (R_{max} - R_0).\left(1 - e^{-(k_{on}c + k_{off})t}\right) + D_1.t$$

(2) $$R_{dis} = R_0 + (R_1 - R_0).e^{-k_{off}t} + D_2.t$$

where c is the protein concentration, t is time, k_{on} is the association rate constant, k_{off} is the dissociation rate constant, D_1 and D_2 are the linear drift terms, and R_{as}, R_{dis}, R_0, R_1, and R_{max} are corresponding changes in the relative resonance signal.

NMR spectroscopy - The families of converged structures for the ACBD3 free Q domain and the ACBD3:PI4KB complex was initially calculated using Cyana 2.1 [3]. The combined automated NOE assignment and structure determination protocol was used to automatically assign the NOE cross-peaks identified in NOESY spectra and to produce preliminary structures. In addition, backbone torsion angle constraints, generated from assigned chemical shifts using the program TALOS+ [4] were included in the calculations. Subsequently, five cycles of simulated annealing combined with redundant dihedral angle constraints were used to produce sets of converged structures with no significant restraint violations (distance and van der Waals violations <0.2Å and dihedral angle constraint violation <5°), which were further refined in explicit solvent using the YASARA software with the YASARA forcefield [5]. The structures with the lowest total energy were selected. Analysis of the family of structures obtained was carried out using the Protein Structure Validation Software suite (www.nesg.org) and Molmol [6]. The statistics for the resulting structures are summarized in SI Table 1.

Giant Unilamellar Vesicle Preparation and Imaging – Giant Unilamellar Vesicles (GUVs) of the desired composition were prepared by electroformation. 50 µg of the lipid mixture was applied on each electrode (5 x 5 cm ITO coated glass) and dried in vacuum overnight. The next day the coated glasses were moved to a home-made teflon chamber and 5 mL of 600 mM sucrose heated to 60 °C was added. Altering current with a maximum amplitude of 1V and frequency of 10 Hz was applied for 1 hour while keeping the chamber at 60 °C. For imaging 100 µL of GUVs and 100 µL of buffer (50 mM Tris pH = 8, 300 mM NaCl, 1 mg/mL BSA) containing appropriate proteins were mixed. The ATTO647N-DOPE and Alexa488 or CFP (mCerulean) labeled proteins were excited simultaneously by 640 nm and 488 nm or 405 nm lasers and imaged using a Zeiss LSM780 confocal microscope.

Supplementary Figures

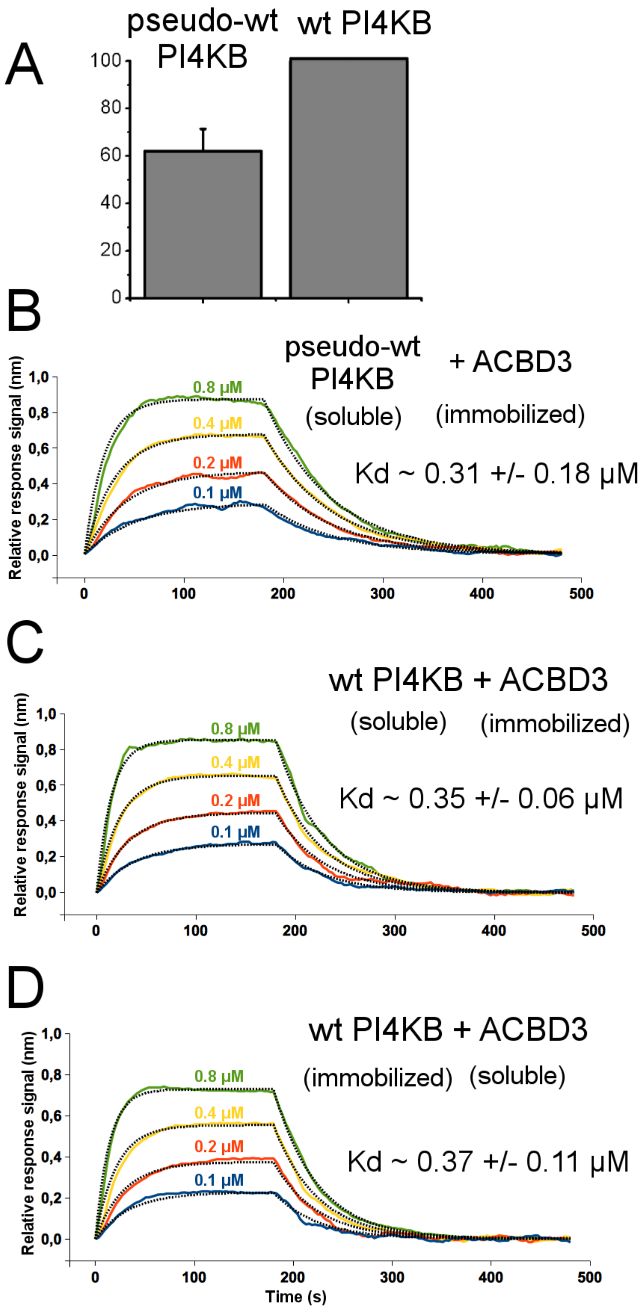

SI Figure 1 – Deletion of the intrinsically disordered loop does not significantly affect neither the enzymatic activity of PI4KB nor its binding to ACBD3

A) **Micelles-based kinase assay** – PI in TX100 micelles was used in a luminescent kinase assay and the production of PI4P was measured. Bar graph presents the mean values of PI4P generated in the presence of the pseudo-wt PI4KB (i.e. PI4KB with the intrinsically disordered loop 423-522 deleted) normalized to the amount of PI4P generated by the full length wt PI4KB. Error bar is a standard error of the mean (SEM) based on two independent experiments.

B) **SPR analysis of the pseudo-wt PI4KB binding to immobilized ACBD3**. Sensorgrams for four concentrations of PI4KB are shown. Same experiment as shown in Fig. 1D except that it was performed on the same chip (in parallel) as experiment in SI Fig. 1C.

C) **SPR analysis of the full length wt PI4KB binding to immobilized ACBD3**. Sensorgrams for four concentrations of PI4KB are shown.

D) **SPR analysis of the full length wt PI4KB (immobilized) binding to soluble ACBD3**. Sensorgrams for four concentrations of PI4KB are shown.

ACBD3

residue numbers start	end	time points (s) 10	20	60	120	600	1800	3600	sequence
241	247	64,9	68,4	69,2	69,0	68,6	68,4	68,4	(M)QQKQQIM
248	253	58,0	58,7	63,6	62,3	63,2	61,6	63,7	AALNSQ
248	255	57,3	60,3	66,3	65,7	66,1	64,5	65,9	AALNSQTA
254	258	43,8	50,6	61,7	67,0	68,8	68,8	68,6	TAVQF
256	258	25,4	32,3	57,0	64,2	70,6	67,6	66,9	VQF
259	261	8,4	16,5	38,8	50,3	60,2	60,2	60,0	QQY
262	269	26,6	29,4	39,4	49,5	72,0	73,6	73,6	AAQQYPGN
262	273	35,0	38,4	47,8	55,2	72,5	74,8	74,2	AAQQYPGNYEQQ
270	276	5,8	7,6	16,7	24,3	47,3	60,2	65,5	YEQQQIL
272	276	1,1	0,9	4,3	8,2	35,7	52,0	60,3	QQQIL
275	280	4,0	4,2	4,5	7,0	27,8	48,4	58,8	ILIRQL
277	284	6,9	10,2	19,5	26,8	48,7	62,7	68,1	IRQLQEQH
281	288	7,4	14,2	36,7	51,2	64,6	64,6	64,3	QEQHYQQY
288	292	21,5	36,3	55,2	59,1	59,9	60,1	59,7	YMQQL
292	296	59,5	62,3	63,3	62,6	62,6	62,4	62,2	LYQVQ
292	297	65,9	68,2	68,7	68,8	68,4	67,2	68,2	LYQVQL
293	297	69,5	72,6	73,0	73,1	72,9	72,6	72,4	YQVQL
293	306	76,0	76,7	77,0	76,9	76,1	76,1	76,0	YQVQLAQQQAALQK
293	308	78,0	77,5	78,7	77,8	76,9	75,4	75,5	YQVQLAQQQAALQKQQ
297	301	71,2	72,8	72,9	71,8	72,0	71,9	72,3	LAQQQ
297	302	72,6	73,7	73,2	73,3	72,5	72,2	72,4	LAQQQA
297	304	70,9	71,1	71,6	71,2	70,5	70,2	69,8	LAQQQAAL
297	306	75,0	75,3	75,3	74,9	74,4	73,9	73,9	LAQQQAALQK
297	308	75,7	76,0	75,9	75,3	74,3	73,9	73,7	LAQQQAALQKQQ
298	308	75,1	75,7	75,6	74,6	73,5	73,2	73,1	AQQQAALQKQQ
302	308	72,6	74,0	73,0	71,8	71,3	70,3	70,2	AALQKQQ

PI4KB

residue numbers start	end	time points (s) 10	20	60	120	600	1800	3600	sequence
1	3	62,0	62,9	63,2	62,8	63,0	62,6	63,1	(AVA)MGD
4	16	73,6	74,7	74,0	73,1	72,1	71,2	71,0	TVVEPAPLKPTSE
4	29	73,7	74,4	73,8	71,8	70,2	69,4	68,6	TVVEPAPLKPTSEPTSGPPGNNGGSL
4	30	74,0	74,9	73,9	72,7	71,0	70,1	69,6	TVVEPAPLKPTSEPTSGPPGNNGGSLL
4	32	74,2	75,1	73,9	73,0	71,0	70,1	69,9	TVVEPAPLKPTSEPTSGPPGNNGGSLLSV
17	29	65,3	65,4	64,0	62,5	60,5	59,4	58,8	PTSGPPGNNGGSL
17	30	66,7	67,3	66,2	64,6	62,7	61,9	61,3	PTSGPPGNNGGSLL
31	36	66,6	68,6	68,8	68,3	68,4	68,3	68,7	SVITEG
31	39	68,6	69,6	69,5	69,0	68,7	68,6	69,0	SVITEGVGE
31	40	71,5	72,4	72,2	71,1	71,7	71,1	71,5	SVITEGVGEL
32	39	63,5	63,8	63,6	62,9	62,5	62,3	62,4	VITEGVGE
33	40	62,1	62,5	62,1	61,7	61,3	61,5	61,3	ITEGVGEL
40	44	63,4	65,2	64,2	63,6	64,5	63,5	63,8	LSVID
40	48	69,0	70,5	70,3	70,0	69,5	69,3	69,2	LSVIDPEVA
40	49	70,1	71,3	71,2	70,9	70,5	70,2	70,2	LSVIDPEVAQ
40	50	72,4	73,8	73,7	73,2	72,9	72,6	72,6	LSVIDPEVAQK
40	54	75,1	77,1	76,6	76,5	76,0	74,3	76,1	LSVIDPEVAQKACQE
40	54	75,3	75,4	75,2	75,1	74,6	74,8	74,3	LSVIDPEVAQKACQE
40	68	76,3	77,9	77,4	77,3	76,6	76,3	76,2	LSVIDPEVAQKACQEVLEKVKLLHGGVAV
40	68	76,6	78,0	77,1	77,8	76,7	77,0	76,2	LSVIDPEVAQKACQEVLEKVKLLHGGVAV
41	50	69,4	70,4	71,0	70,2	70,0	69,7	69,5	SVIDPEVAQK
41	54	72,6	73,7	74,2	72,9	73,6	72,9	72,9	SVIDPEVAQKACQE
41	54	72,3	73,5	73,2	72,8	72,8	72,6	72,2	SVIDPEVAQKACQE
42	50	65,9	67,4	67,1	66,5	66,4	66,3	66,4	VIDPEVAQK
45	50	65,6	68,0	67,4	66,4	67,1	67,3	66,5	PEVAQK
45	54	70,9	71,4	72,5	72,0	72,1	69,9	71,6	PEVAQKACQE
45	54	71,1	72,0	72,0	71,5	71,8	71,4	71,8	PEVAQKACQE
55	60	63,6	64,4	64,4	63,9	64,1	64,0	64,1	VLEKVK
55	68	64,3	65,1	65,9	64,8	64,2	63,2	63,0	VLEKVKLLHGGVAV
57	68	58,3	61,0	63,6	62,9	63,1	63,4	63,9	EKVKLLHGGVAV
58	68	57,1	60,1	62,7	62,5	62,3	62,1	62,3	KVKLLHGGVAV
59	68	55,0	58,8	61,7	61,5	61,5	61,1	61,5	VKLLHGGVAV
61	68	49,9	54,3	57,9	57,7	56,9	56,8	57,1	LLHGGVAV

Scale

[%]

0
10
20
30
40
50
60
70
80

SI Figure 2 – Peptides used in the HDX-MS analysis of the ACBCD3 Q domain:PI4KB N-terminal region complex. The residue start number, residue end number, the percentage of hydrogens exchanged for deuteria at 7 different time points, and the peptide sequence are displayed for every peptide. The numbers are also color coded, 0% exchange is in blue and 80% would correspond to red.

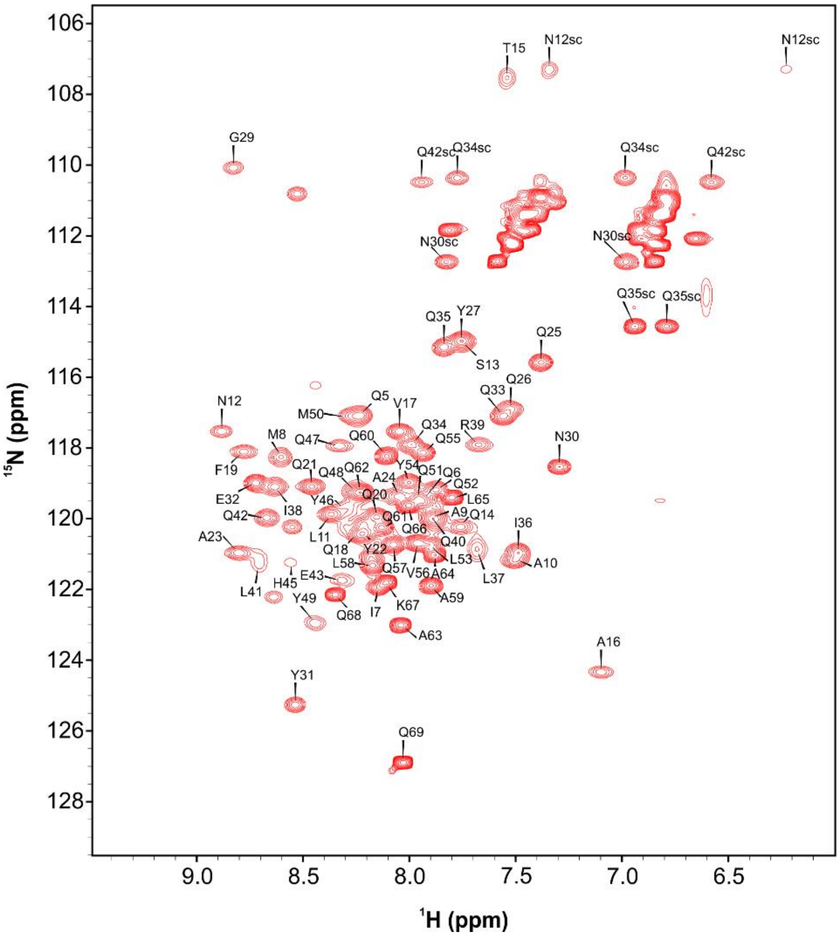

SI Figure 3 – 2D 15N/1H HSQC spectrum for the free ACBD3 Q-domain. The backbone amide group signals are labeled with the respective residue codes. The side-chain amides that could be unambiguously assigned are labeled with the residue codes and 'sc' abbreviation.

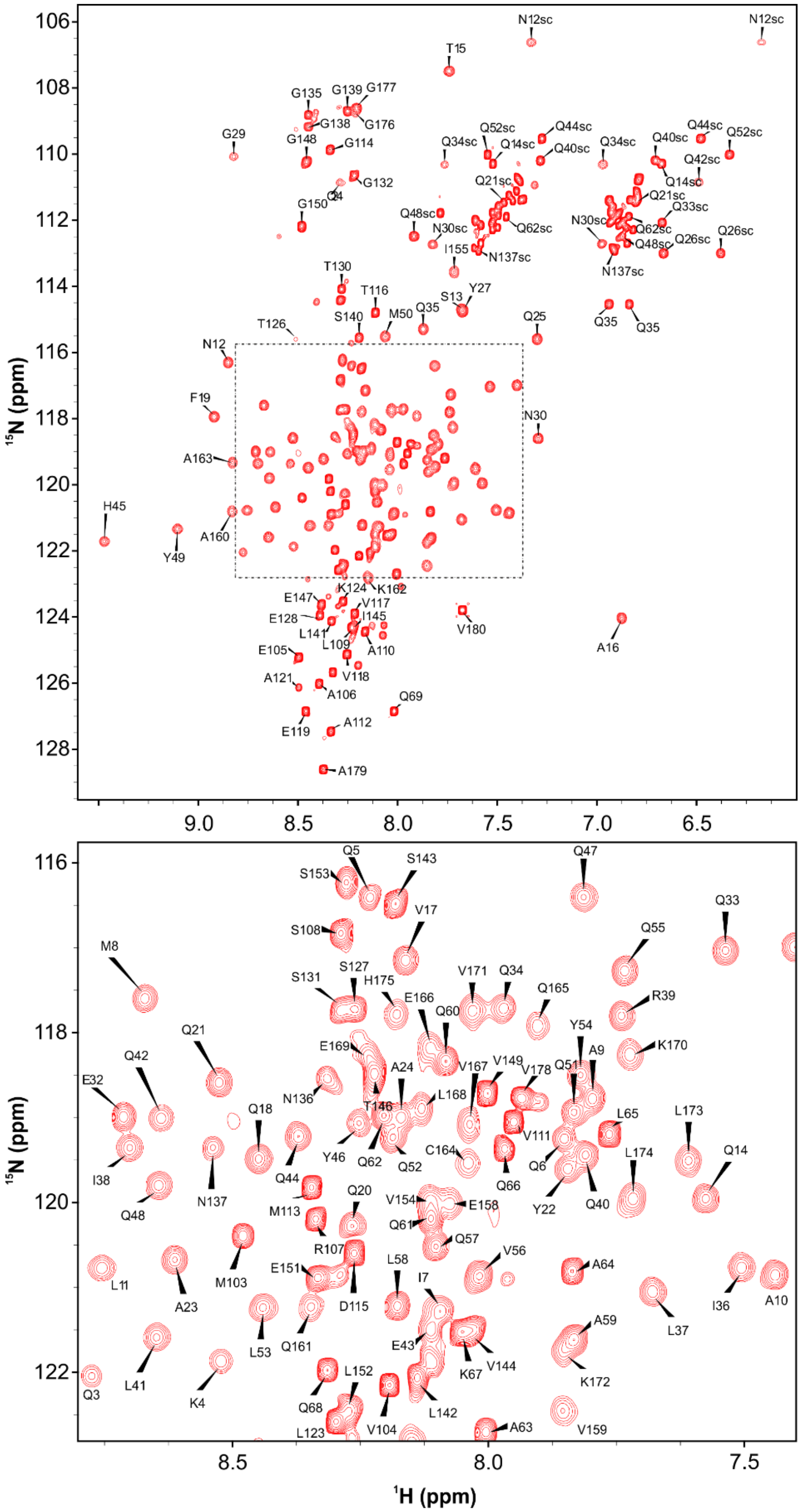

SI Figure 4 – 2D 15N/1H HSQC spectrum for the ACBD3 Q domain:PI4KB N-terminal region protein complex. Both backbone and side-chain (the ‘sc’ abbreviation) amide group signals are labeled with the respective residue codes. The residue numbers for the PI4KB N-terminal region protein are incremented by a 100. Top: the full spectrum, bottom: the highly overlapped central region.

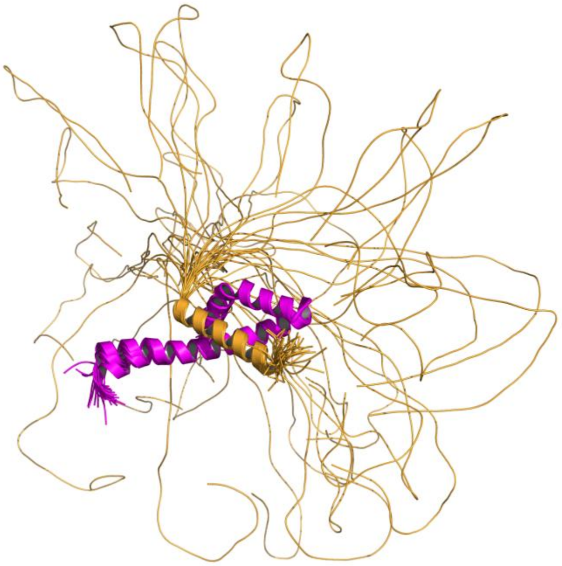

SI Figure 5 - The overall structure of the ACBD3 Q domain in complex with the PI4KB N-terminal region. Superposition of 30 converged structures. PI4KB is in orange, ACBD3 Q domain in magenta.

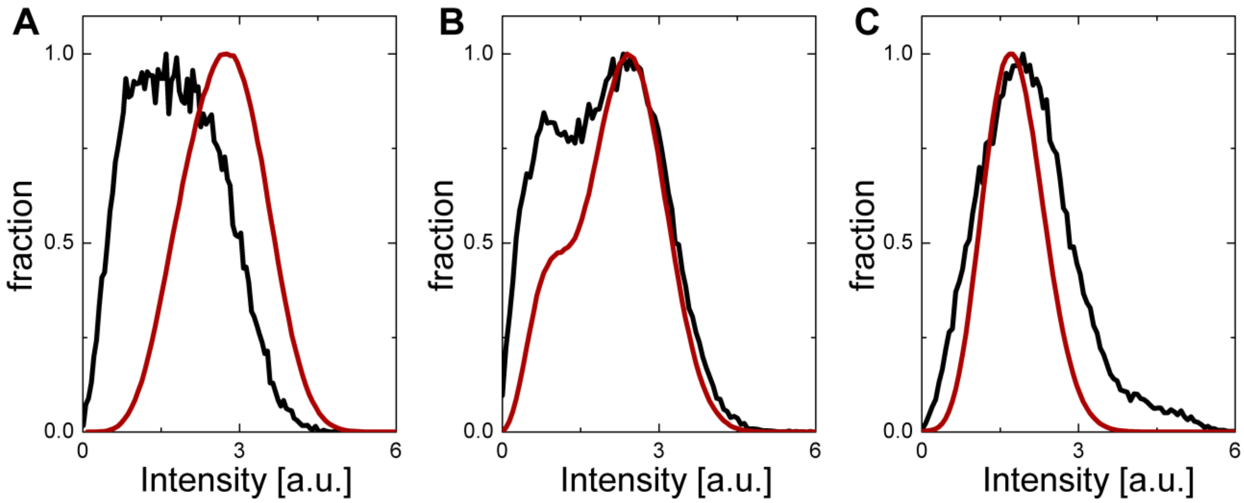

SI Figure 6 – Histograms of fluorescence intensities of the PI4P reporter (CFP-SidC). Intensities in the CFP channel (excitation at 405 nm, emission collected between 465-571 nm) of the background pixels (outside GUVs, red line) and the membrane pixels (selected by the fluorescence signal of the membrane reporter Atto647-DOPE acquired in separate channel, excitation at 633 nm, emission collected between 645-759 nm, black line) are compared under three different protein/ATP conditions: A) PI4KB, ACBD3, but no ATP, a system referring to no phosphorylation. Pixels comprising fluorescence signal from the membrane and its vicinity have lower intensity than the background pixels as there is no reporter signal on the membrane and in the lumen of GUVs B) **PI4KB + ATP** C) **PI4KB + ACBD3 + ATP**.

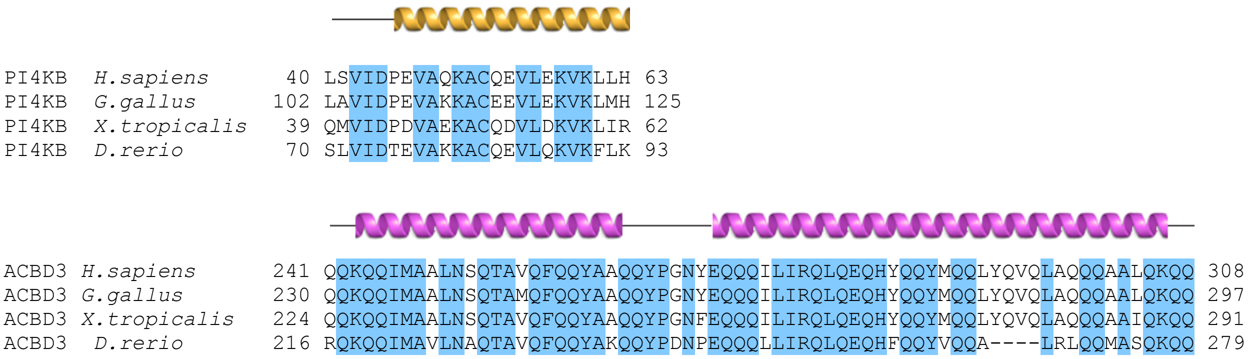

SI Figure 7 - Multiple alignment of the PI4KB and ACBD3 interacting domains. Sequences were obtained from Genbank and aligned using the ClustalX algorithm. Blue areas represent conserved amino acid residues; the numbers indicate amino acid residue positions. The secondary structures present in the NMR structure of the complex of PI4KB and ACBD3 are indicated in orange and magenta, respectively.

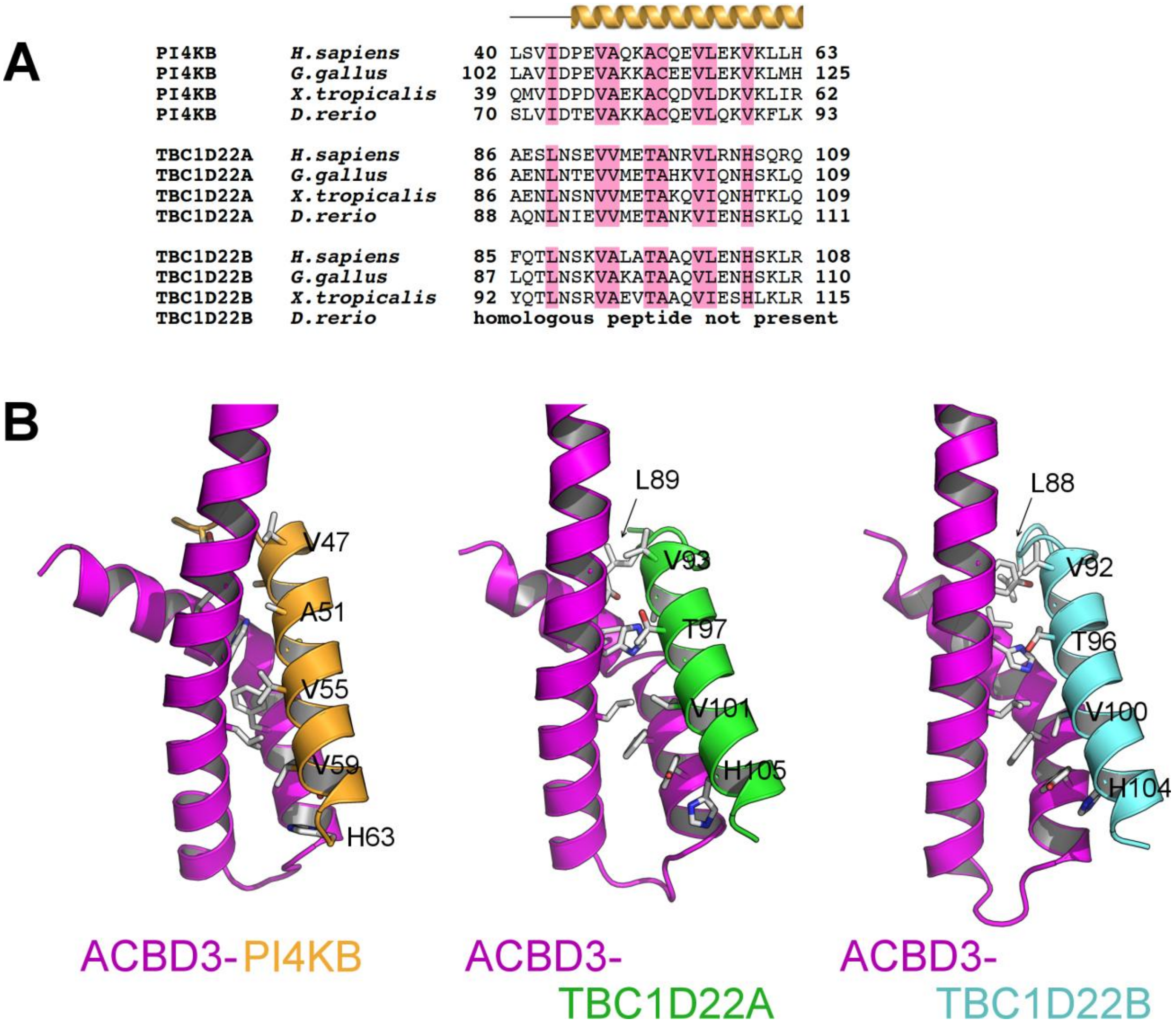

SI Figure 8 - Homology models of the ACBD3-TBC1D22A and ACBD3-TBC1D22B complexes

A) **Multiple alignments of the PI4KB and TBC1D22A/B regions interacting with the ACBD3 Q-domain.** Sequences were obtained from Genbank and aligned using the ClustalX algorithm. Red areas represent amino acid residues present at the ACBD3-PI4KB and putative ACBD3-TBC1D22A/B interfaces; the numbers indicate amino acids positions. B) **Detailed view of the ACBD3-TBC1D22A/B complexes**. Homology models of the ACBD3-TBC1D22A/B complexes were generated using the I-TASSER software [7] and our NMR spectroscopy-based structure of the ACBD3-PI4KB complex as a template**.** ACBD3 is shown in magenta, PI4KB in orange, TBC1D22A in green, and TBC1D22B in aquamarine.

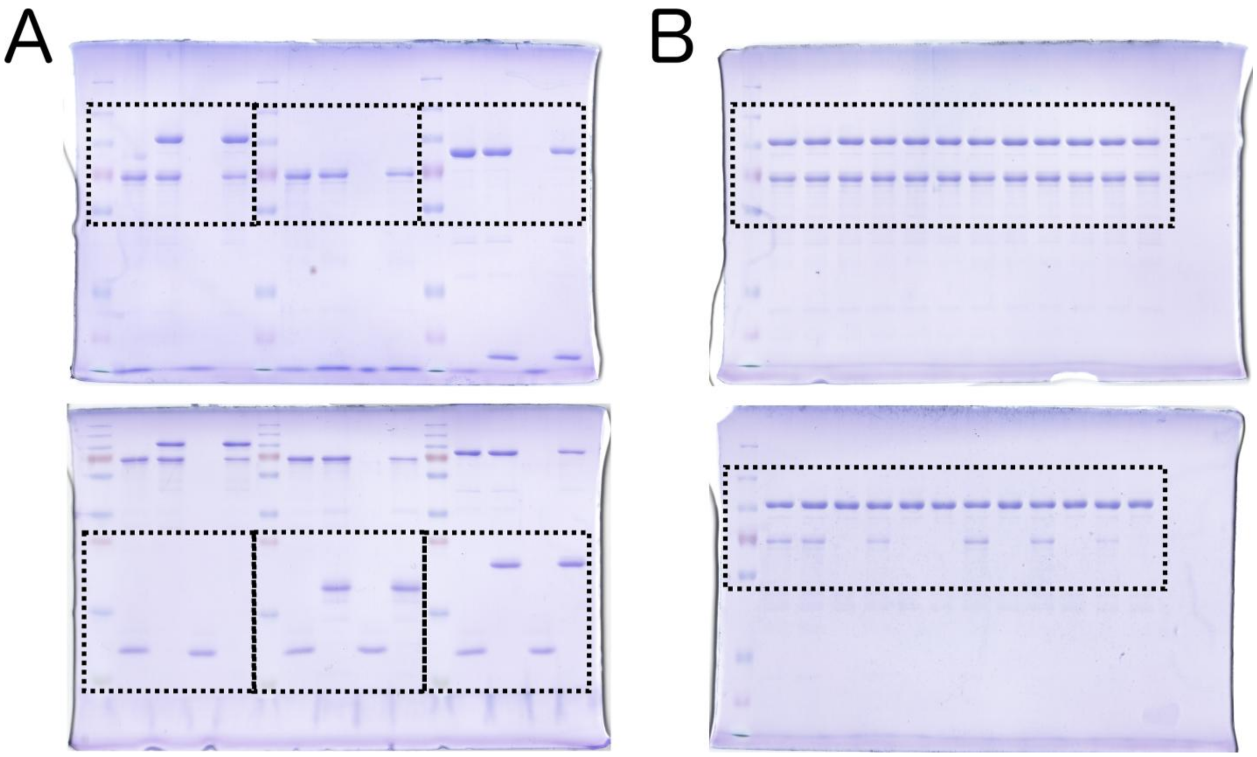

SI Figure 9 – Full length gels

A) **Full length gel accompanying Figure 1** – Dashed rectangles are shown in Fig. 1

B) **Full length gel accompanying Figure 2** – Dashed rectangles are shown in Fig. 2

Video legends

1) **Video 1: Mitochondria recruitment experiment – wt Q domain**. Cells transfected with AKAP1-FRB-CFP, GFP-PI4KB and wild-type Q domain-FKBP-mRFP constructs filmed during addition of rapamycin. AKAP1-FRB-CFP is localized on the mitochondria. Note that when rapamycin is added (time 1 min 15 s) the wild-type Q domain-FKBP-mRFP rapidly translocates to mitochondria as well and is followed with ~ 2 min delay by the GFP-PI4KB.
2) **Video 2:Mitochondria recruitment experiment – H^{264}A Q domain**. The same experiment as in video 1 performed using the H^{264}A Q domain mutant. Note that the mutant Q domain translocates to the mitochondria as well but is not able to recruit the GFP-PI4KB.

Supplementary Tables

SI Table 1 – **Structural statistics for the final water-refined sets of structures of the Q domain and the kinase helix**

	ACBD3/PI4KB		*ACBD3*	
Non-redundant distance and angle constrains				
Total number of NOE constraints	2396		1086	
Short-range NOEs				
Intra-residue (i = j)	605		282	
Sequential (| i - j | = 1)	694		311	
Medium-range NOEs (1 < | i - j | < 5)	656		332	
Long-range NOEs (| i - j | ≥ 5)	441		161	
Torsion angles	158		118	
Hydrogen bond restrains	-		-	
Total number of restricting constraints	2554		1204	
Total restricting constraints per restrained residue	17.9		18.0	
Residual constraint violations				
Distance violations per structure				
0.1 – 0.2 Å	15.22		12.2	
0.2 – 0.5 Å	7.31		6.23	
> 0.5 Å	0		0	
r.m.s. of distance violation per constraint	0.03 Å		0.04 Å	
Maximum distance violation	0.50 Å		0.50 Å	
Dihedral angle violations per structure				
1 – 10 °	2.11		2.10	
> 10 °	0		0	
r.m.s. of dihedral violations per constraint	0.38 °		0.41 °	
Maximum dihedral angle violation	4.90 °		4.9 °	
Ramachandran plot summary from Procheck				
Most favoured regions	95.1 %		94.3 %	
Additionally allowed regions	4.9 %		5.5 %	
Generously allowed regions	0.0 %		0.2 %	
Disallowed regions	0.0 %		0.0 %	
r.m.s.d. to the mean structure	*ordered*[1]	*all residues*	*ordered*[2]	*all residues*
All backbone atoms	0.4 Å	10.9 Å	0.8 Å	1.3 Å

All heavy atoms	0.8 Å	11.0 Å	1.2 Å	1.8 Å

[1] residues 5A-66A,54B-75B, [2] residues 5A-66ASI

SI Table 2 - **List of the DNA constructs used in experiments**.

**Expression of ACBD3 in *E. coli***
pRSFD - His_6 - GB1 - TEV site - ACBD3 (residues 1-528)
pRSFD - His_6 - GB1 - TEV site - ACBD3 (residues 241-308)
pRSFD - His_6 - GB1 - TEV site - ACBD3 (residues 1-528, Phe258Ala)
pRSFD - His_6 - GB1 - TEV site - ACBD3 (residues 1-528, Tyr266Ala)
pRSFD - His_6 - GB1 - TEV site - ACBD3 (residues 1-528, His284Ala)
pRSFD - His_6 - GB1 - TEV site - ACBD3 (residues 1-528, Tyr285Ala)
pRSFD - His_6 - GB1 - TEV site - ACBD3 (residues 1-528, Tyr288Ala)
**Expression of PI4KB in *E. coli***
pRSFD - His_6 - GB1 - TEV site - PI4KB (residues 1-423 & 522-816)
pRSFD - His_6 - GB1 - TEV site - PI4KB (residues 1-68)
pRSFD - His_6 - GB1 - TEV site - PI4KB (residues 1-423 & 522-816, Val42Ala)
pRSFD - His_6 - GB1 - TEV site - PI4KB (residues 1-423 & 522-816, Ile43Ala)
pRSFD - His_6 - GB1 - TEV site - PI4KB (residues 1-423 & 522-816, Val47Ala)
pRSFD - His_6 - GB1 - TEV site - PI4KB (residues 1-423 & 522-816, Val55Ala)
pRSFD - His_6 - GB1 - TEV site - PI4KB (residues 1-423 & 522-816, Leu56Ala)
**Other expression in *E. coli***
pRSFD - His_6 – TEV site - CFP – SidC (residues 578-913)
**Expression in mammalian cells**
pGFP - PI4KB (residues 1-816)
pCFP - FRB (residues 2021-2113) - AKAP1 (residues 34-63)
pmRFP - FKBP12 (residues 3-109) - ACBD3 (residues 241-308)
pmRFP - FKBP12 (residues 3-109) - ACBD3 (residues 241-308, His284Ala)
pGFP - ACBD3 (residues 241-308)

AKAP1, A kinase anchor protein 1; CFP, cyan fluorescent protein (mCerulean); GFP, enhanced green fluorescent protein; mRFP, monomeric far red fluorescent protein; FKBP12, FK506 binding protein 12; FRB, fragment of mTOR that binds rapamycin; GB1, B1 domain of Streptococcal protein G; TEV, Tobacco etch virus protease cleavage site.

Supplementary references

1 Greninger, A. L., Knudsen, G. M., Betegon, M., Burlingame, A. L. & DeRisi, J. L. ACBD3 interaction with TBC1 domain 22 protein is differentially affected by enteroviral and kobuviral 3A protein binding. *MBio* **4**, e00098-00013, doi:10.1128/mBio.00098-13 (2013).

2 Kay, B. K., Thai, S. & Volgina, V. V. High-throughput biotinylation of proteins. *Methods Mol Biol* **498**, 185-196, doi:10.1007/978-1-59745-196-3_13 (2009).

3 Herrmann, T., Guntert, P. & Wuthrich, K. Protein NMR structure determination with automated NOE assignment using the new software CANDID and the torsion angle dynamics algorithm DYANA. *J Mol Biol* **319**, 209-227 (2002).

4 Shen, Y., Delaglio, F., Cornilescu, G. & Bax, A. TALOS+: a hybrid method for predicting protein backbone torsion angles from NMR chemical shifts. *J Biomol NMR* **44**, 213-223, doi:10.1007/s10858-009-9333-z (2009).

5 Harjes, E. *et al.* GTP-Ras disrupts the intramolecular complex of C1 and RA domains of Nore1. *Structure* **14**, 881-888, doi:10.1016/j.str.2006.03.008 (2006).

6 Koradi, R., Billeter, M. & Wuthrich, K. MOLMOL: a program for display and analysis of macromolecular structures. *J Mol Graph* **14**, 51-55, 29-32 (1996).

7 Yang, J. *et al.* The I-TASSER Suite: protein structure and function prediction. *Nat Methods* **12**, 7-8, doi:10.1038/nmeth.3213 (2015).
